# Clinical determinants of recurrence in pTa bladder cancer following transurethral resection of bladder tumor

**DOI:** 10.1186/s12885-022-09733-8

**Published:** 2022-06-08

**Authors:** Seung-hwan Jeong, Jang Hee Han, Chang Wook Jeong, Hyeon Hoe Kim, Cheol Kwak, Hyeong Dong Yuk, Ja Hyeon Ku

**Affiliations:** 1 Department of Urology, Seoul National University Hospital, Seoul, Korea; 2 Department of Urology, Seoul National University College of Medicine, Seoul, Korea

**Keywords:** Ta bladder cancer, Recurrence, TURB

## Abstract

**Background:**

Non-muscle invasive bladder cancer can be controlled by transurethral resection of bladder (TURB), but suffers from frequent recurrences in 60–70% of cases. Although, recurrence interval after TURB influences treatment course and prognosis, its implication and risk factors have not been fully elucidated. We evaluated the risk factors of early (within 1 yr) and late (after 1 yr) recurrence of pTa bladder cancer and clinical significance of recurrence interval on disease progression and overall survival.

**Methods:**

In this study, pTa bladder cancer patients enrolled in prospective patient registry system of Seoul National University, SUPER-UC, were retrospectively examined to determine the clinical risk factors for recurrence and its significance regarding to recurrence interval. A total of 1067 bladder cancer patients who underwent TURB between March 20 and June 2021 were included and classified into three groups of no recurrence, early, or late recurrence to be comparatively analyzed.

**Results:**

Early recurrence was associated with poorer cystectomy-free survival and overall survival than late recurrence. Risk factors for early recurrence included a high number of previous TURB, tumor multiplicity, tumor location, tumor shape, incompleteness of TURB, and high tumor grade. Otherwise, late recurrence was associated with low-grade tumors with insufficient TURB depth.

**Conclusion:**

Patients with risk factors for early recurrence should be closely followed up with special cautions.

## Introduction

Bladder cancer accounts for 3% of diagnosed cancers worldwide and is more prevalent in developed countries, with the sixth highest cancer incidence in the USA [1]. The most critical barometer of bladder cancer is muscle invasiveness, which determines the clinical course, including treatment option and prognosis. Muscle invasive bladder cancer (MIBC) mandates aggressive treatments such as radical cystectomy or concurrent chemoradiotherapy [2].

Almost 75% of bladder cancers present as non-muscle invasive bladder cancer (NMIBC) with limited invasion to the mucosal (Ta and CIS) or submucosal layer (T1) [3]. NMIBC is initially controlled through transurethral resection of bladder tumor (TURB), followed by regular cystoscopy owing to frequent recurrence as high as 60–70% [4]. NMIBC can be classified into low, intermediate, high, and very high-risk groups based on risk factors, including pT stage, concomitant CIS, histologic grade, patient’s age, multiplicity of tumor, and tumor size [3]. Tumor progression was variable between the risk groups as the 5-year progression rate was less than 1% in the low-risk group and as high as 40% in the very high-risk group [5].

Specifically, Ta stage cancer accounts for 60% of NMIBC cases, while T1 and CIS cancers account for 30% and 10%, respectively [4]. Patients with Ta low-grade tumors experience recurrence in 70% and progression in 5% of cases, whereas Ta high grade progresses in 30–40% of cases [6]. However, risk classifications in Ta bladder cancer remain in the gray zone; these risks are related to early or late recurrence after TURB, possibly influencing the clinical course of cancer progression. In this study, we analyzed the clinical parameters influencing the recurrence of Ta bladder cancer following TURB and classified them as involved with early or late recurrence based on the recurrence duration of 1 year which was the median duration of recurrence.

## Materials and methods

### Patient selection and stratification

We reviewed the clinical data of patients enrolled in the Seoul National University Prospective Enrolled Registry for urothelial cancer (SUPER-UC) from March 2016 to June 2021 under approval of institutional review board (IRB No. 2111–110-1272). SUPER-UC enrolled 1915 patients with general information such as underlying disease; medication; operation history; and pre-, intra-, and postoperative data including laboratory data, pathologic results, and clinical data regarding tumor recurrence or progression [7]. A total of 1067 patients underwent TURB in SUPER-UC, and their registered data were queried after obtaining permission of the institutional review board. Patients were followed up every 3 months for a year after TURB. Patients were stratified into three groups according to recurrence: no recurrence, early recurrence, and late recurrence, based on the recurrence period of 1 year after the latest TURB.

### TURB procedure

TURB was performed by experienced surgeons with intention to complete resection ensuring free surgical margins under natural optic view. Bladder tumor recurrence was evaluated through cystoscopic examination and confirmed by pathologic result of TURB. Intravesical BCG treatment was done in 2.41%, 3.85%, and 4.52% in no-, early-, and late- recurrence group respectively without statistical significance.

### Statistical analysis 

One-way ANOVA test was utilized for the analysis of parametric values, and the chi-square test was used for categorical variables. Kaplan–Meier survival analysis was adopted for determining cystectomy-free survival, and overall survival analysis with the log-rank test was used to determine significance. Cox regression analysis was performed to reveal the factors associated with early or late recurrence compared with no recurrence. The analysis was performed using SPSS 25 (IBM) and Prism 9 (GraphPad) software. Statistical significance was set at *P* < 0.05.

## Results

### Patient characteristics

The institutional review board was appropriately achieved for this study (IRB no. 2111–110-1272). Patients were classified into three groups according to recurrence after TURB: no recurrence, early recurrence, and late recurrence. Early and late recurrence were defined as recurrence within and after 1 year of TURB, respectively. Among the 1067 patients, 704 had no recurrence, 208 had early recurrence, and 155 had late recurrence (Table 1). Average age and sex were similar between the three groups as well as underlying diseases and performance status represented by obesity, diabetes mellitus, hypertension, and Eastern Cooperative Oncology Group grade (Table 1).

**Table 1:** Demographic data of Ta bladder cancer patients

	No (*n* = 704)	Early (*n* = 208)	Late (*n* = 155)	*p* value
Age	68.6	69.2	68.8	0.745
Sex				
Male	583 (82.8)	162 (77.9)	127 (81.9)	0.27
Female	121 (17.2)	46 (22.1)	28 (18.1)	
BMI	24.6	24.3	24.4	0.615
DM	153 (21.7)	49 (23.6)	35 (22.6)	0.851
HTN	326 (46.3)	103 (49.5)	67 (43.2)	0.487
ECOG grade				
0	691 (98.2)	204 (98.1)	151 (97.4)	0.771
1	11 (1.6)	4 (1.9)	4 (2.6)	
2	2 (0.3)	0	0	

*BMI* body mass index, *DM* Diabetes mellitus, *HTN* hypertension

### Operative findings and pathologic results related to recurrence

The number of previous TURB cases was higher in the early recurrence group than in the no recurrence and late recurrence groups. In particular, previous TURB number 3 or more in early recurrence was 1.8-fold higher than that in no recurrence (10.1% vs 5.7%, *p* = 0.01). Tumor multiplicity over 7 was also most frequent in early recurrence (no vs. early vs. late: 14.2% vs 26.6% vs 19.2%, *p* < 0.001). Furthermore, the following factors were associated with early recurrence: tumor location in the posterior or both lateral walls, sessile or flat erythematous tumor shape, incomplete TURB, and high-grade histology (Table 2). Insufficient resection depth, which does not include the muscle layer and low-grade tumors, was associated with late recurrence. Large tumors measuring > 3 cm and concomitant CIS lesions tended to be higher in early recurrence but without significance.

**Table 2:** Characteristics of Ta bladder cancer in each group

	No (*n* = 704)	Early (*n* = 208)	Late (*n* = 155)	*p* value
Previous TURB				
0	478 (67.9)	115 (55.3)	95 (61.3)	0.01
1—2	186 (26.4)	72 (34.6)	49 (31.6)	
≥ 3	40 (5.7)	21 (10.1)	11 (7.1)	
Number of mass				
1	310 (46.0)	65 (32.7)	57 (37.7)	< 0.001
2—7	268 (39.8)	81 (40.7)	65 (43.0)	
≥ 8	96 (14.2)	53 (26.6)	29 (19.2)	
Mass size				
< 3 cm	536 (79.3)	155 (76.7)	123 (80.9)	0.607
> = 3 cm	140 (20.7)	47 (23.3)	29 (19.1)	
Tumor location				
Trigone	162 (23.0)	60 (28.8)	41 (26.5)	0.196
Posterior wall	195 (27.7)	87 (41.8)	51 (32.9)	0.001
Both lateral	416 (59.1)	144 (69.2)	91 (58.7)	0.025
Anterior	143 (20.3)	53 (25.5)	37 (23.9)	0.229
Dome	144 (20.5)	37 (17.8)	29 (18.7)	0.66
Prostatic urethra	27 (3.8)	10 (4.8)	6 (3.9)	0.817
Tumor shape				
Papillary	629 (93.5)	178 (88.1)	143 (94.1)	0.03
Sessile/Flat erythematous	44 (6.5)	24 (11.9)	9 (5.9)	
Resection depth				
Submucosa	15 (2.2)	4 (2.0)	10 (6.5)	0.039
Muscle	676 (97.5)	198 (97.5)	142 (92.8)	
Perivesical fat	2 (0.3)	1 (0.5)	1 (0.7)	
Complete TURB				
No	9 (1.3)	10 (4.9)	4 (2.6)	0.009
Yes	683 (98.7)	196 (95.1)	150 (97.4)	
Grade				
Low grade	329 (46.7)	99 (47.6)	95 (61.3)	0.004
High grade	375 (53.3)	109 (52.4)	60 (38.7)	
Concomitant CIS	40 (5.7)	16 (7.7)	5 (3.2)	0.188

### Laboratory findings related to recurrence

Preoperative laboratory data were analyzed to gain further insight into factors related to recurrence. Complete blood count and inflammatory markers, including ESR and CRP, were similar among the three groups (Table 3). Notably, the neutrophil-to-lymphocyte ratio (NLR) was 2.76 in early recurrence, which was higher than that in patients with no recurrence or late recurrence (2.16 and 1.97, *p* = 0.005). This finding is in concordance with previous reports that denoted high NLR as an adverse factor for recurrence and disease progression in NMIBC [8]. Moreover, severe hematuria (red blood cell [RBC] count > 50/high power field [HPF]) was also reported the most in early recurrence, in line with a previous report identifying gross hematuria as a risk factor for recurrence and progression in NMIBC [9].

**Table 3:** Preoperative laboratory data

	Non (*n* = 704)	Early (*n* = 208)	Late (*n* = 155)	*p* value
WBC	6.38	6.35	6.26	0.818
Hb	13.43	13.2	13.4	0.295
Plt	222.8	216.1	223.0	0.531
Seg. Neutrophil	57.5	59.1	56.2	0.111
Lymphocyte	30.9	29.3	32.2	0.088
ANC	3741	3774	3463	0.233
NLR	2.16	2.76	1.97	0.005
ESR	20.9	24.3	21.6	0.431
CRP	0.339	0.338	0.309	0.973
Urine cytology				
Negative	471 (82.2)	127 (77.4)	108 (86.4)	0.254
Atypical	96 (16.8)	33 (20.1)	15 (12.0)	
Malignant	6 (1.0)	4 (2.4)	2 (1.6)	
Urine WBC				
0	453 (79.5)	124 (82.1)	105 (82.7)	0.854
1 + 2 +	98 (17.2)	26 (17.2)	20 (15.7)	
3 +	19 (3.3)	5 (3.3)	2 (1.6)	
Urine RBC				
< 5	399 (66.5)	98 (58.7)	98 (73.1)	0.055
< 20	73 (12.2)	27 (16.2)	9 (6.7)	
< 50	31 (5.2)	8 (4.8)	10 (7.5)	
> = 50	97 (16.2)	34 (20.4)	17 (12.7)	

*WBC* white blood cell, *Hb* hemoglobin, *Plt* platelet, *Seg. Neurophil* segmented neutrophil, *ANC* absolute neutrophil count, *NLR* neutrophil-to-lymphocyte ratio, *ESR* erythrocyte sedimentation rate, *CRP* C-reactive protein, *RBC* red blood cell

### Survival analysis

**Fig. 1:**
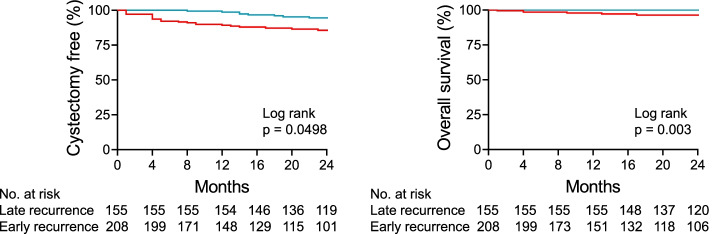
Cystectomy-free and Overall survival in early and late recurrence patients. Red and green lines represent early and late recurrence respectively

The high-risk progression of NMIBC should be managed via radical cystectomy. Thus, cystectomy-free survival reflects tumor progression and is a therapeutic goal to be attained in terms of bladder-sparing treatment [10]. Two-year cystectomy-free survival in early recurrence was 85.6% and 94.6% in late recurrence (*p* = 0.0498). Moreover, the overall survival in years in early recurrence was 96.4%, which is lower than the 100% in late recurrence (*p* = 0.003) (Fig. 1). To reveal the relevant variables associated with early and late tumor recurrence, Cox proportional hazards regression analyses were conducted on the variables such as number of mass, mass size, tumor shape, resection depth, completeness of TURB, tumor grade, and concomitant CIS comparing no recurrence versus early or late recurrences. Multiplicity of tumor (HR 1.43 for 2—7 tumors, *p* = 0.035, HR 2.266 for ≥ 8 tumors, *p* < 0.001) and sessile tumor shape (HR 1.928, *p* = 0.002) were significantly associated with early tumor recurrence (Table 4). Furthermore, complete TUR reduced early recurrence (HR 0.338, *p* = 0.003). Meanwhile, multiplicity of tumor (HR 1.441 for 2–7 tumors, *p* = 0.044, HR 2.242 for ≥ 8 tumors, *p* < 0.001) was associated with late tumor recurrence. In addition, high grade tumors recurred less when comparing no recurrence versus late recurrence (HR 0.697, *p* = 0.034) (Table 5).

**Table 4:** Multivariate COX regression analysis comparing no recurrence and early recurrence

	HR (95% CI)	*p* value
Number of mass		
1	Reference	< 0.001
2—7	1.43 (1.026 – 1.994)	0.035
≥ 8	2.266 (1.553 – 3.306)	< 0.001
Mass size		
< 3 cm	Reference	
> = 3 cm	1.08 (0.768 – 1.519)	0.657
Tumor shape		
Papillary	Reference	
Sessile/Flat erythematous	1.928 (1.284 – 2.894)	0.002
Resection depth		
Submucosa	Reference	0.615
Muscle	1.576 (0.498 – 4.989)	0.439
Perivesical fat	2.915 (0.296 – 28.699)	0.359
Complete TURB		
No	Reference	
Yes	0.338 (0.164 – 0.698)	0.003
Grade		
Low grade	Reference	
High grade	0.966 (0.722 – 1.291)	0.813
CIS	1.005 (0.536 – 1.887)	0.987

**Table 5:** Multivariate COX regression analysis no comparing no recurrence and late recurrence

	HR (95% CI)	*p* value
Number of mass		
1	Reference	0.002
2—7	1.441 (1.01 – 2.056)	0.044
≥ 8	2.242 (1.436 – 3.502)	< 0.001
Mass size		
< 3 cm	Reference	
> = 3 cm	0.97 (0.64 – 1.472)	0.887
Tumor shape		
Papillary	Reference	
Sessile/Flat erythematous	0.86 (0.412 – 1.795)	0.688
Resection depth		
Submucosa	Reference	0.277
Muscle	0.597 (0.308 – 1.158)	0.127
Perivesical fat	1.001 (0.124 – 8.07)	0.999
Complete TURB		
No	Reference	
Yes	1.313 (0.407 – 3.144)	0.814
Grade		
Low grade	Reference	
High grade	0.697 (0.5 – 0.973)	0.034
CIS	0.654 (0.237 – 1.804)	0.412

## Discussion

Patients with bladder cancer experience various clinical courses, from initial cure via TURB to disease progression requiring radical cystectomy or systematic chemotherapy [11]. Although TURB and intravesical instillation treatment provide a durable cure rate, bladder cancer recurs in up to 80% of cases [12]. Furthermore, recurrence within 1 year is an intermediate risk factor for low-grade NMIBC in AUA risk stratification [2]. In the present study, we demonstrated the clinical factors associated with recurrence after TURB and classified them into three groups according to the recurrence interval after TURB. The European Association of Urology stratified risk groups into low-, intermediate-, high-, and very high-risk groups. The risk factors comprising the stratifications included patient age of 70 years, pathologic T stage, histologic grade, tumors measuring > 3 cm, tumor multiplicity, and concomitant CIS [3]. The easily accessible web-based stratification tool (https://nmibc.net/) provides stratified risk groups and progression probabilities based on the suggested factors. Moreover, the NCCN guidelines for bladder cancer also stratify low-risk, intermediate-risk, and high-risk groups with stratification factors similar to those of the EAU guidelines. We demonstrated that recurrence within 1 year after TURB threatens cystectomy-free survival and overall survival, which are critical factors for the risk classification of NMIBC. Notably, frequent recurrence was associated with early recurrence, implying that repetitive recurrence shortens the recurrence interval and worsens tumor progression. Anne Sörenby et al., reported that completeness of TURB by experienced surgeon is important for early recurrence of bladder cancer after TURB [13]. Interestingly, tumor location in the posterior or both lateral walls was associated with early recurrence. Several studies have reported that tumor location in the bladder is associated with lymph node metastasis and a higher pathologic stage after radical cystectomy [14]. In addition, a preclinical study demonstrated high vasculature in the inferolateral aspect of the bladder; this is associated with enhanced lymphovascular invasion of tumors in this location [15].

NLR reflects systematic inflammatory conditions that are critical for the prognosis of MIBC and high-risk NMIBC [16, 17]. High NLR is associated with tumor recurrence after TURB in NMIBC [18]. One study reported that an NLR over 2.5 is associated with worse recurrence free survival in NMIBC; in our study, average NLR of early recurrence was 2.76, exceeding 2.5, whereas it was less in the other two groups [19]. Hematuria is known to be a significant risk factor for progression-free and overall survival in bladder cancer but has not been utilized for risk classification in the EAU guidelines [20]. Our data also showed that significant hematuria (> 50 RBC/HPF) was associated with early recurrence.

The prognosis of late recurrence was more favorable than that of early recurrence in terms of cystectomy-free survival and overall survival. Low-grade tumors and insufficient TURB depth were associated with late recurrence. It is unclear whether insufficient resection depth is a risk factor for late recurrence since local fulguration is allowed for very small lesions in low-grade tumors. However, even in low-grade tumors, sufficient resection depth should be ensured to avoid late recurrence. The multivariate COX regression analysis revealed that number of tumor was a risk factor for early and late recurrence, consistent with risk classification according to tumor multiplicity by EAU guideline.

Our study has limitations stemming from its retrospective study design and short follow-up period. However, in an effort to elaborate data confidence, we utilized prospective cohort data to minimize errors or missing values. We suggest that recurrence interval and number should be utilized in risk classifications, and extra effort should be made to conduct complete TURB with sufficient resection depth in any case.

## Data Availability

The datasets generated and/or analysed during the current study are not publicly available due to the clinical data are regulated strictly by our IRB, but are available from the corresponding author on reasonable request.
